# Immunohistochemical expression of SMAD4, CK19, and CA19-9 in fine needle aspiration samples of pancreatic adenocarcinoma: Utility and potential role

**DOI:** 10.1186/1742-6413-4-13

**Published:** 2007-06-22

**Authors:** Mauricio Zapata, Cynthia Cohen, Momin T Siddiqui

**Affiliations:** 1 Department of Pathology and Laboratory Medicine, Emory University Hospital, Atlanta, GA, USA

## Abstract

**Background:**

Pancreatic adenocarcinoma comprises 85% of all cases of pancreatic malignancies. From a diagnostic standpoint, these tumors are readily diagnosed by fine needle aspiration, with an accuracy of greater than 90%; however it is often difficult to ascertain whether these are primary or metastatic in nature. This study was undertaken to see the usefulness of CK19, CA19-9 and a newly described marker, SMAD4 in confirming the pancreatic origin of these tumors. Briefly, SMAD4 (DPC4) is a tumor-suppressor gene located on chromosome 18q which has been shown to mediate the downstream effects of TGF-β superfamily signaling, resulting in growth inhibition. The loss of SMAD4, which as been reported to occur in 55% of pancreatic ductal adenocarcinomas may lead to up regulation of cell cycle proteins and hence increase cellular proliferation. In addition, SMAD4 has been suggested to possibly have prognostic potential, with the presence of SMAD4, indicating shorter survival after resection.

**Design:**

Clinical data was reviewed to identify patients with proven, primary pancreatic adenocarcinoma. A total of 25 patients with diagnostic material from fine needle aspiration cell blocks, were retrieved from our files at Emory University Hospital. In addition cell blocks from clinically diagnosed non-pancreatic adenocarcinomas were also selected as controls for this study (10 cases of colonic adenocarcinoma, 10 cases of pulmonary adenocarcinoma, 10 cases of breast ductal carcinoma and 10 cases of ovarian mucinous adenocarcinoma). Formalin fixed, paraffin-embedded sections from these were stained with SMAD4, CK19, and CA19-9, using pressure cooker antigen retrieval, labeled polymer HRP (DAKO), and the DAKO autostainer.

**Results:**

Immunohistochemical staining was reviewed based on intensity (negative, low-positive, and high-positive) and percentage of cells. In primary pancreatic ductal adenocarcinoma, CK 19 showed diffuse cytoplasmic positivity in 23 of 25 cases, CA 19-9 showed apical cytoplasmic staining in all 25 cases, and SMAD4 showed nuclear staining in 20 of 25 cases. In the control group comprising of non-pancreatic adenocarcinoma SMAD4 was negative (100%) in all 10 cases of colonic and pulmonary adenocarcinoma. However 1 of 10 cases (10%) of breast and ovarian adenocarcinoma did show low positivity nuclear staining. However the expression of CA19-9 and CK19 was more variable in these different non-pancreatic malignancies.

**Conclusion:**

Pancreatic adenocarcinoma showed positive immunohistochemical staining for SMAD4 in 80%, CK19 in 100% and CA19-9 in 100% of the selected cases. These markers, when used as a panel, may confirm the diagnosis of pancreatic adenocarcinoma in fine needle aspiration samples, and help in differentiating from metastatic adenocarcinoma. This may help in determination of appropriate surgical and chemotherapeutic options.

## Background

Neoplasia of the pancreas consists of a wide spectrum of benign and malignant tumors, with pancreatic ductal adenocarcinoma comprising 85% of malignant cases. The American Cancer Society estimates that 33,730 new cases of pancreatic cancer will be diagnosed in 2006; and 32,300 fatalities will be attributed to the disease [1]. Although rates of pancreatic cancer have slowly declined in the United States over the past 15–25 years, it is the fourth leading cause of cancer mortality [1], with a 5-year survival rate of as low as 5% [2]. The poor prognosis of pancreatic ductal adenocarcinoma is mainly attributed to its insidious and inconspicuous growth often presenting late in the clinical disease process. It is estimated that at initial diagnosis of disease, approximately 50% of patients will have distant metastases, while only 10% will have tumors localized to the pancreas [3].

The incidence of pancreatic ductal adenocarcinoma increases gradually with age, with approximately 80% of cases diagnosed in individuals 60–80 years [4]. Males are more likely than females to be diagnosed with pancreatic cancer; and ethnic disparities exist, with African and Japanese Americans having higher incidence and mortality rates as compared to Anglo Americans [5]. Although the exact cause of pancreatic cancer remains unknown, several environmental and host factors have been shown to be associated with the progression to malignant disease. Among the risk factors investigated, smoking is the most significant with smokers having a two-fold increased risk of developing pancreatic cancer as compared to non-smokers [6].

It is clinically very important, that a correct and accurate diagnosis of primary pancreatic ductal adenocarcinoma be rendered, and may require immunohistochemical stains to exclude other known primaries. We selected SMAD4, CA19-9 and CK 19 immunohistochemical stains to help us in this endeavor. All three markers are expressed in pancreatic adenocarcinoma and are uniformly negative in pancreatic non-ductal neoplasms [7,8]. hence, they as a panel may be very helpful in diagnosing pancreatic ductal carcinoma, which is the premise and hypothesis for our study.

## Methods

A retrospective review of clinical data was evaluated through a search of Emory University Hospital Laboratory information systems to identify patients with proven, primary pancreatic adenocarcinoma. A total of twenty-five patients with diagnostic material from CT guided fine needle aspiration, cell blocks collected from January 1, 2002 through December 31, 2005 were retrieved. All cases were clinically a part of the Pancreatic tumor registry, with clinically proven pancreatic adenocarcinoma, based on combined clinical/radiologic and histologic data. In addition cell blocks from clinically diagnosed non-pancreatic adenocarcinomas were also selected as controls for this study (10 cases of colonic adenocarcinoma, 10 cases of pulmonary adenocarcinoma, 10 cases of breast ductal carcinoma and 10 cases of ovarian mucinous adenocarcinoma). All sections from the cells blocks were, formalin-fixed, paraffin-embedded tissue (5 microns), and were tested for the presence of SMAD4 (1:40, Novacastra, Newcastle on Tyne), CK19 (1:50, DAKO, Carpinteria, CA), and CA 19-9 (1:200, Novacastra, Newcastle on Tyne), using a horseradish peroxidase (HRP) labeled polymer and heat-induced antigen retrieval. The DAKO ENVISION system was used, which consists of a two step horseradish peroxidase labeled polymer conjugated with secondary antibodies (DAKO ENVISION System, DAKO Corp., Carpintera, CA). This method was used in combination with the automated DAKO AUTOSTAINER (DAKO Corp.). Hematoxylin was used as counter stain. Negative controls were performed and consisted of primary antibody replaced by buffer specific antibody absorbed with antigen.

Sections were deparfinnized and rehydrated. Antigen retrieval was performed in citrate buffer (pH 6) using an electric pressure cooker for 5 minutes at 120°C with cooling for 10 minutes before immunostaining. All tissues are then exposed to 3% hydrogen peroxide for 5 minutes, primary antibody for 30 minutes, labeled polymer HRP for 30 minutes, diaminobenzidine as chromogen for 5 minutes and DAKO automation hematoxylin as counter stain for 15 minutes. All incubation steps were performed at room temperature and between incubations, sections were washed with Tris-buffered saline (TBS) solution. Cover slipping was performed using the Tissue-Tek SCA (Sakura Finetek USA, Inc., Torrance, CA) automatic coverslipper.

## Results

Immunohistochemical staining, with appropriate positive and negative controls, were reviewed independently by two pathologists (MTS and CC). No interobserver discrepancy was noted. A total of 25 pancreatic adenocarcinomas were reviewed, of which 20 cases were poorly differentiated adenocarcinoma, while 5 were moderately differentiated adenocarcinoma. The reviewing pathologists evaluated the tissue sections based on both the staining intensity and percentage positivity of cells. The intensity was graded as negative (no staining observed), low-positive (moderate degree of staining), and high-positive (strong intense staining). There were twenty-five cases, 80% (20 of 25 cases) were positive for SMAD4, while 100% of the cases were positive for both CK 19 and CA19-9. These findings are summarized in Table 1. SMAD4 staining is nuclear, with high staining intensity observed in 11 (44%) of the cases. No cytoplasmic staining for SMAD4 was noted. Diffuse cytoplasmic staining was noted for CK19 and a high staining intensity was observed in 23 (92%) of the cases studied. CA19-9 displayed an apical cytoplasmic staining and a high staining intensity was observed in all 25 (100%) of the cases. Lastly, benign pancreatic ductal epithelial cells were identified in 21 of 25 cases, benign pancreatic acinar cells were identified in 9 of 25 cases, and benign pancreatic islet cells were noted in 11 of 25 cases and were uniformly negative for SMAD4, CA19-9 and CK 19 immunohistochemical staining. In view of these results the sensitivity for SMAD4 for pancreatic ductal adenocarcinoma is 80% while for CA19-9 and CK19 it is 100%. The specificity for all three markers is 0%.

**Table 1:** SMAD4, CK 19 and CA 19-9 in pancreatic tumor cell immunoreactivity is highlighted in this table.

	SMAD4	CK19	CA19-9
Staining Pattern	Nuclear	Diffuse Cytoplasmic	Apical Cytoplasmic
Negative	5 cases	0 cases	0 cases
Low Positive	9 cases	2 cases	0 cases
High Positive	11 cases	23 cases	25 cases
% Range of Positive cells	Low Positive 60–80% & High Positive 40–90%	Low Positive 80–90% & High Positive 95–100%	High Positive 95–100%

The results of the control group comprising of non-pancreatic adenocarcinoma are summarized in Table 2. SMAD4 was negative (100%) in all 10 cases of colonic and pulmonary adenocarcinoma. However, 1 of 10 cases (10%) of breast and ovarian adenocarcinoma did show low positivity nuclear staining. The expression of CA19-9 and CK19 was more variable in these different non-pancreatic malignancies and is further tabulated in Table 2.

**Table 2:** SMAD4, CK 19 and CA 19-9 immunoreactivity in non-pancreatic adenocarcinoma is highlighted in this table.

	SMAD4	CK19	CA19-9
Colorectal adenocarcinoma	0 of 10 cases	4 of 10 cases	6 of 10 cases
Pulmonary adenocarcinoma	0 of 10 cases	5 of 10 cases	3 of 10 cases
Breast ductal carcinoma	1 of 10 cases	7 of 10 cases	3 of 10 cases
Ovarian mucinous adenocarcinoma	1 of 10 cases	8 of 10 cases	7 of 10 cases

## Discussion

In 2006, a total of 33,730 new cases of pancreatic cancer were identified according to American Cancer Society statistics [1]. In addition, for that same year the estimated deaths from pancreatic cancer were 32,300 individuals making it the fourth leading cause of cancer mortality [1]. Overall, these tumors are associated with a poor prognosis, and several studies have shown that the best predictor of prolonged survival is complete surgical resection. Host factors play an integral role in the development of pancreatic carcinoma. It is now well accepted that pancreatic carcinoma follows a progression of histological and molecular changes leading to the development of malignancy. Analogous to the sequence of colorectal carcinoma from non-neoplastic epithelium to adenoma to invasive carcinoma, pancreatic carcinoma also follows a similar paradigm. It has been demonstrated that precursor lesions known as pancreatic intraepithelial neoplasias (PanIN) are associated with increasing degrees of cytological and architectural atypia associated with the accumulation of genetic alterations in cancer associated genes, ultimately leading to malignancy. Among these genetic alterations, K-ras activation and HER-2/neu expression appears to occur relatively early, while p16 inactivation occurs at an intermediate stage, and ultimately p53, BRCA2, and SMAD4 (DPC4) inactivation occurs late in the disease process [9].

SMAD4 or DPC4 (deleted in pancreatic cancer, locus 4) is a tumor suppressor gene located on the long arm of chromosome 18. It was first described in pancreatic cancer by Harn et al. in 1996, who demonstrated that nearly 90% of pancreatic cancers show loss of heterozygosity for this gene [10,11]. SMAD4 has also been suggested to be genetically responsible for familial juvenile polyposis and also has been implicated as a late genetic event in colorectal carcinogenesis [12]. SMAD4 is part of a group of SMAD proteins that are an essential component of the TGFβ signaling pathway, which negatively regulates the growth of epithelial cells. More specifically, SMAD4 binds to other SMAD proteins forming a complex, which interacts with DNA binding proteins leading to regulation of transcription and ultimately decreased cellular proliferation [12]. Thus, the loss of SMAD4 expression in pancreatic ductal adenocarcinoma leads to upregulation of cell cycle proteins and hence increases cellular proliferation.

CA19-9 or carbohydrate antigen 19-9 is a blood group related antigen and is biochemically related to the Lewis A blood group substance. CK19 is a cytokeratin which has been described to show strong immunoreactivity in pancreatic adenocarcinoma in histologic sections, however, its use in cell blocks from patients with pancreatic adenocarcinoma has not been previously described. The purpose of our study was to assess the diagnostic utility of SMAD4 along with CK19 and CA19-9 in confirming the pancreatic origin of tumors.

**Figure 1:**
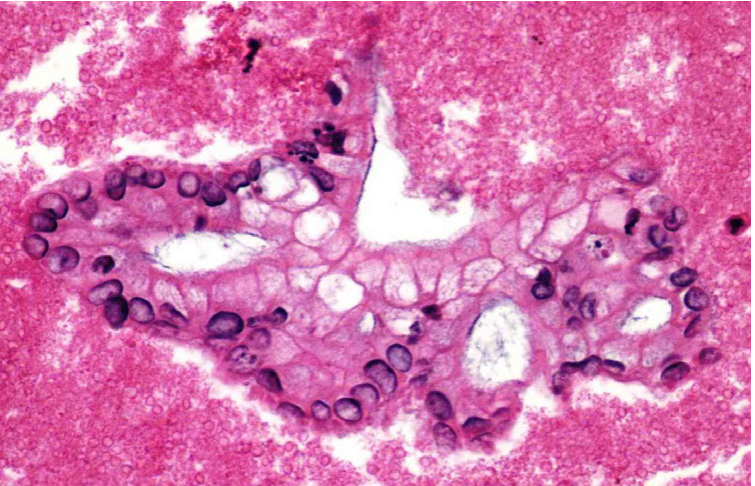
Representative fragment of Pancreatic Adenocarcinoma (H&E, 40×).

**Figure 2:**
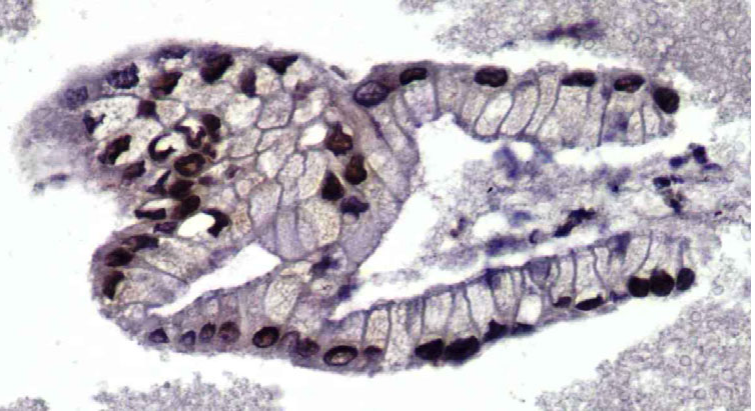
Representative fragment of Pancreatic Adenocarcinoma stained with SMAD4 showing high positivity (IHC, 40×).

Our study has demonstrated that the immunohistochemical staining panel of SMAD4, CK19, and CA19-9 is useful in confirming the diagnosis of pancreatic adenocarcinoma. Twenty-five selected cases of clinically confirmed pancreatic adenocarcinoma (see Figure 1) were evaluated for all three markers, of which 80% of the cases stained positive for SMAD4, while 100% of the cases were positive for CK19 and CA19-9. In regard to SMAD4 immunohistochemical staining properties, our cases, demonstrated a crisp nuclear staining pattern with virtually no cytoplasmic or background staining (Figure 2 and 5). SMAD4 showed a low positivity in 9 (36%) cases (Figure 5), with 60–80% of the tumor cells exhibiting immunoreactivity and the other additional 11 (44%) cases showed a high positivity (Figure 2) with 40–90% of the tumor cells exhibiting immunoreactivity. The non-pancreatic malignancies that were studied as a control group (Table 2) also yielded the data that SMAD4 shows negative immunoreactivity in colonic and pulmonary adenocarcinoma, however in 10% of breast ductal and ovarian mucinous adenocarcinoma, focal low positivity nuclear staining may be observed.

**Figure 3:**
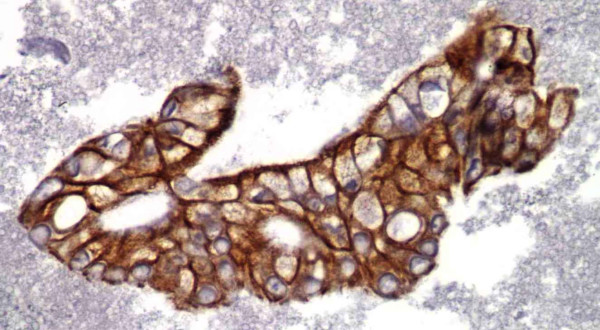
Representative fragment of Pancreatic Adenocarcinoma stained with CK19 (IHC, 40×).

**Figure 4:**
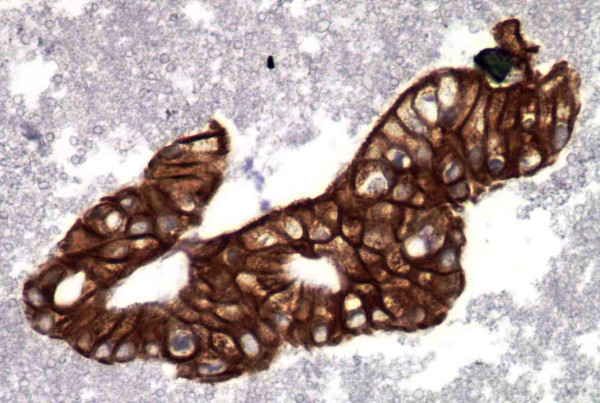
Representative fragment of Pancreatic Adenocarcinoma stained with CA19-9 (IHC, 40×).

**Figure 5:**
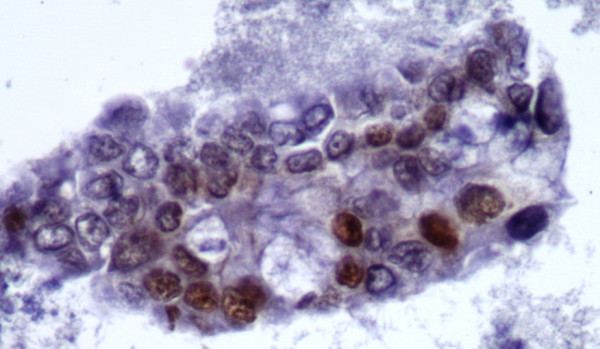
Representative fragment of Pancreatic Adenocarcinoma stained with SMAD4 showing low positivity (IHC, 40×).

The staining pattern for CK19 was diffuse cytoplasmic and noted to be present in 100% of the cases (Figure 3). High staining positivity was observed in 23 (92%) cases. This study also showed that in addition to SMAD4 and CK19, CA19-9 was also useful for confirming the diagnosis of pancreatic adenocarcinoma. CA19-9 showed apical cytoplasmic staining in all 25 (100%) of our cases, with a range of 95–100% of the tumor cells with high positivity (Figure 4). The non-pancreatic control group (Table 2) showed variable staining for both CA19-9 and CK19.

To our knowledge, this is the first time that SMAD4 staining in combination with CK19 and CA19-9 has been evaluated in pancreatic adenocarcinoma. The different staining intensity noted in these three markers may be attributed to labeling of proteins at a different phase of pathogenesis of pancreatic adenocarcinoma. Among other primary pancreatic neoplasm, Cao et al demonstrated that in contrast to pancreatic adenocarcinomas that show loss of SMAD4 protein in 55% of cases, loss of SMAD4 expression is absent in pancreatic nonductal neoplasms [7]. In addition, prior studies have demonstrated the presence of SMAD4 genetic alterations among colorectal carcinoma [12] and cholangiocarcinoma [13] in only 11–17% and 45.2% of cases, respectively. Taken together, these studies suggest that SMAD4 expression is highly useful in diagnosing pancreatic adenocarcinoma.

The concept that SMAD4 expression may correlate with prognosis, is also of interest. Biankin et al. evaluated 348 patients with pancreatic adenocarcinoma and demonstrated that tumor size (greater than 4.5 cm), resection margin involvement, and perineural invasion were independent prognostic factors [14]. Far more interesting however, was the finding that SMAD4 expression within excised pancreatic carcinoma was associated with a worse outcome. They demonstrated that loss of SMAD4 expression was associated with a favorable prognosis (median survival, 13.6 vs 6.4 months; logrank, P = 0.257). The advantage of SMAD4 expression as a prognostic indicator is that it is potentially assessable preoperatively in comparison to tumor size, resection margins, perineural invasion, and lymph node metastasis, which can only be accurately evaluated after surgery. In our own study, 18 of the 20 patients with SMAD4 positivity died within 6 months of diagnosis and 2 (low positivity SMAD4 expression) of the 20 patients were still alive 7 and 9 months after diagnosis of pancreatic adenocarcinoma. The 5 patients who were negative for SMAD4 expression died between 11 and 13 months after diagnosis of pancreatic adenocarcinoma. In view of this data, ultimately in the future, preoperative evaluation of SMAD4 status may help in determining appropriate surgical and chemotherapeutic options.

In summary, our study shows that SMAD4, CK19 and CA19-9 are helpful markers for confirming the diagnosis of primary pancreatic ductal carcinoma. Also, showing that fine needle aspiration is an excellent diagnostic modality for obtaining samples for diagnosing primary malignancies in the pancreas and if need be, usage of the studied immunohistochemical stains would help in confirming this diagnosis.
